# Severe COVID-19 patients exhibit elevated levels of autoantibodies targeting cardiolipin and platelet glycoprotein with age: a systems biology approach

**DOI:** 10.1038/s41514-023-00118-0

**Published:** 2023-08-24

**Authors:** Dennyson Leandro M. Fonseca, Igor Salerno Filgueiras, Alexandre H. C. Marques, Elroy Vojdani, Gilad Halpert, Yuri Ostrinski, Gabriela Crispim Baiocchi, Desirée Rodrigues Plaça, Paula P. Freire, Shahab Zaki Pour, Guido Moll, Rusan Catar, Yael Bublil Lavi, Jonathan I. Silverberg, Jason Zimmerman, Gustavo Cabral-Miranda, Robson F. Carvalho, Taj Ali Khan, Harald Heidecke, Rodrigo J. S. Dalmolin, Andre Ducati Luchessi, Hans D. Ochs, Lena F. Schimke, Howard Amital, Gabriela Riemekasten, Israel Zyskind, Avi Z. Rosenberg, Aristo Vojdani, Yehuda Shoenfeld, Otavio Cabral-Marques

**Affiliations:** 1 Interunit Postgraduate Program on Bioinformatics, Institute of Mathematics and Statistics (IME), University of Sao Paulo (USP), Sao Paulo, SP Brazil; 2 Department of Immunology, Institute of Biomedical Sciences, University of São Paulo, São Paulo, SP Brazil; 3 Regenera Medical 11860 Wilshire Blvd., Ste. 301, Los Angeles, CA 90025 USA; 4 Ariel University, Ari’el, Israel; 5 Zabludowicz Center for Autoimmune Diseases, Sheba Medical Center, Tel-Hashomer, Israel; 6 Saint Petersburg State University Russia, Saint Petersburg, Russia; 7 Department of Clinical and Toxicological Analyses, School of Pharmaceutical Sciences, University of São Paulo, São Paulo, Brazil; 8 Laboratory of Molecular Evolution and Bioinformatics, Department of Microbiology, Biomedical Sciences Institute, University of São Paulo, São Paulo, 05508-000 Brazil; 9 Departament of Nephrology and Internal Intensive Care Medicine, Charité University Hospital, Berlin, Germany; 10 Scakler faculty of medicine, Tel Aviv University, Tel Aviv, Israel; 11 Department of Dermatology, George Washington University School of Medicine and Health Sciences, Washington, DC USA; 12 Maimonides Medical Center, Brooklyn, NY USA; 13 Department of Structural and Functional Biology, Institute of Biosciences, São Paulo State University (UNESP), Botucatu, São Paulo, Brazil; 14 Institute of Pathology and Diagnostic Medicine, Khyber Medical University, Peshawar, Pakistan; 15 CellTrend Gesellschaft mit beschränkter Haftung (GmbH), Luckenwalde, Germany; 16 Bioinformatics Multidisciplinary Environment, Federal University of Rio Grande do Norte, Natal, Brazil; 17 Department of Biochemistry, Federal University of Rio Grande do Norte, Natal, Brazil; 18 Department of Clinical and Toxicological Analyses, Federal University of Rio Grande do Norte, R.N., Natal, Brazil; 19 Department of Pediatrics, University of Washington School of Medicine, and Seattle Children’s Research Institute, Seattle, WA USA; 20 Department of Medicine B, Sheba Medical Center, Tel Hashomer, Israel; 21 Sackler Faculty of Medicine, Tel-Aviv University, Tel-Aviv, Israel; 22 Department of Rheumatology, University Medical Center Schleswig-Holstein Campus Lübeck, Lübeck, Germany; 23 Department of Pediatrics, NYU Langone Medical Center, New York, NY USA; 24 Department of Pathology, Johns Hopkins University, Baltimore, MD USA; 25 Department of Immunology, Immunosciences Laboratory, Inc., Los Angeles, CA USA; 26 Cyrex Laboratories, LLC 2602 S. 24th St., Phoenix, AZ 85034 USA; 27 Department of Pharmacy and Postgraduate Program of Health and Science, Federal University of Rio Grande do Norte, Natal, Brazil; 28 Department of Medicine, Division of Molecular Medicine, University of São Paulo School of Medicine, São Paulo, Brazil; 29 Laboratory of Medical Investigation 29, University of São Paulo School of Medicine, São Paulo, Brazil; 30 Network of Immunity in Infection, Malignancy, Autoimmunity (NIIMA), Universal Scientific Education and Research Network (USERN), São Paulo, SP Brazil

**Keywords:** Infectious diseases, Risk factors

## Abstract

Age is a significant risk factor for the coronavirus disease 2019 (COVID-19) severity due to immunosenescence and certain age-dependent medical conditions (e.g., obesity, cardiovascular disorder, and chronic respiratory disease). However, despite the well-known influence of age on autoantibody biology in health and disease, its impact on the risk of developing severe COVID-19 remains poorly explored. Here, we performed a cross-sectional study of autoantibodies directed against 58 targets associated with autoimmune diseases in 159 individuals with different COVID-19 severity (71 mild, 61 moderate, and 27 with severe symptoms) and 73 healthy controls. We found that the natural production of autoantibodies increases with age and is exacerbated by SARS-CoV-2 infection, mostly in severe COVID-19 patients. Multiple linear regression analysis showed that severe COVID-19 patients have a significant age-associated increase of autoantibody levels against 16 targets (e.g., amyloid β peptide, β catenin, cardiolipin, claudin, enteric nerve, fibulin, insulin receptor a, and platelet glycoprotein). Principal component analysis with spectrum decomposition and hierarchical clustering analysis based on these autoantibodies indicated an age-dependent stratification of severe COVID-19 patients. Random forest analysis ranked autoantibodies targeting cardiolipin, claudin, and platelet glycoprotein as the three most crucial autoantibodies for the stratification of severe COVID-19 patients ≥50 years of age. Follow-up analysis using binomial logistic regression found that anti-cardiolipin and anti-platelet glycoprotein autoantibodies significantly increased the likelihood of developing a severe COVID-19 phenotype with aging. These findings provide key insights to explain why aging increases the chance of developing more severe COVID-19 phenotypes.

## Introduction

There is increasing evidence connecting coronavirus disease 2019 (COVID-19), caused by the severe acute respiratory syndrome virus 2 (SARS-CoV-2), with underlying autoimmune pathology^1,2^. The triggers of this intersection between COVID-19 and autoimmunity have been ascribed to exacerbated and chronic inflammation^3^, e.g., by promoting the exposure to self-antigens and activation of bystander T cells caused by systemic high cytokine levels^4^, and due to the molecular mimicry between SARS-CoV-2 spike and human proteins^5–8^. Patients with severe COVID-19 develop profound organ damage due to a combination of several autoinflammatory and autoimmune responses, causing, among others, myopathy^9^, vasculitis, arthritis, antiphospholipid syndrome (APS)^10^ associated with deep vein thrombosis, pulmonary embolism, and stroke, as well as other organ damage to lungs, kidneys, and those forming the neurological system^11,12^. Furthermore, immune dysregulation is a hallmark of post-COVID syndrome^13^ causing heterogeneous symptoms such as fatigue, vascular dysfunction, pain syndromes, neurological manifestations, and neuropsychiatric syndromes^14–17^.

Following the initial discovery of autoantibodies against type I interferons (IFNs) in patients with life-threatening COVID-19^18^, several reports documented elevated levels of autoantibodies targeting various additional cytokines and chemokines and their receptors^19^, but also cardiac antigens^20^, G protein-coupled receptors (GPCR), renin-angiotensin system (RAS)-related molecules, and those against anti-cardiolipin^21–27^, ribosomal P proteins, chromatin proteins, thyroid antigens^28^, anti-nuclear antigen (ANA)^28,29^, and anti-neutrophil cytoplasmic proteins (ANCA)^30^ in patients with severe SARS-CoV-2 infections. We recently reported a large spectrum of autoantibodies linked to autoimmune diseases that associate with COVID-19 severity^31^. Autoantibody levels often accompany anti-SARS-CoV-2 antibody concentrations as essential predictors of COVID-19 outcome^31^. However, the impact of the aging effect on autoantibody levels was barely explored in these studies.

Notably, aging has been strongly associated with increased morbidity and mortality of elderly patients with SARS-CoV-2 infections^32–34^. Elderly individuals present an increased risk of developing autoimmune diseases for several reasons. For instance, immunosenescence and its associated immune dysregulation^35–37^, increased amounts of free DNA in the blood circulation^38^, and enhanced serum levels of autoantibodies^39,40^. In this context, considering the well-known effect of age on autoantibody biology and immune pathophysiology in health and disease^1,3,11,41–43^, to understand better the particular influence of age on autoantibodies induced by SARS-CoV-2 could provide new insights into the COVID-19 pathophysiology and development of severe phenotypes as well as the autoantibody biology. To address this issue, we performed a follow-up systems immunology analysis of our recent cross-sectional study of 159 individuals with different COVID-19 outcomes (mild, moderate, and severe) compared to 73 healthy controls^44,45^.

## Results

### Age-dependent increase of autoantibody levels in severe COVID-19 patients

**Fig. 1:**
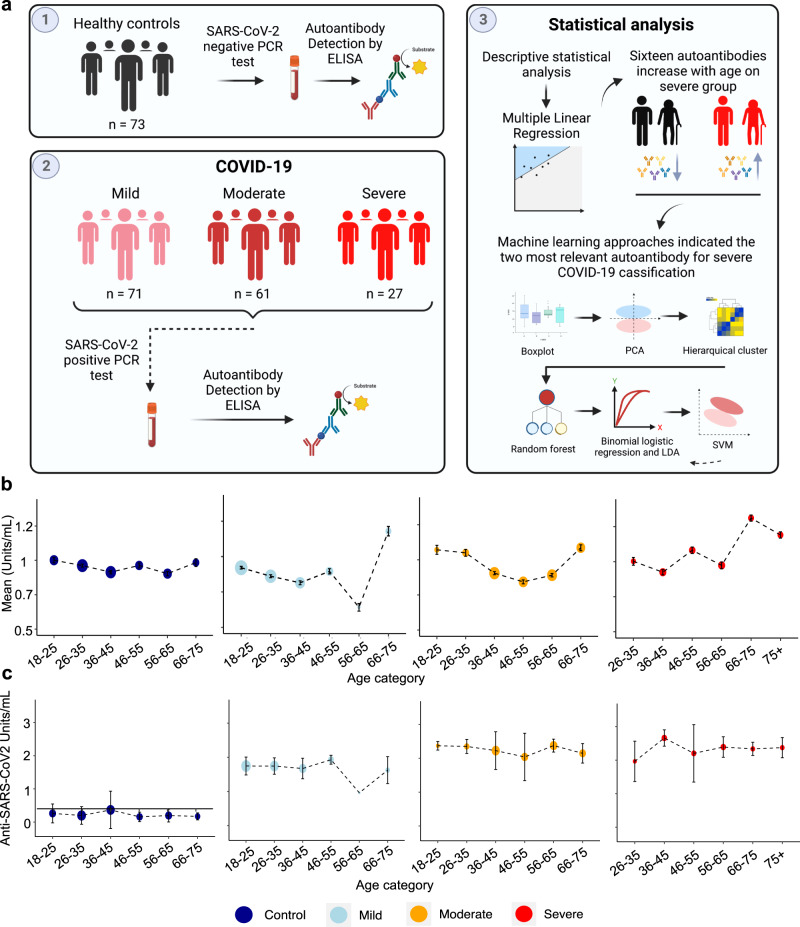
The systems immunology approach identifies an aging effect on autoantibody levels of COVID-19 patients. **a** Following sample acquisition, bioinformatics, and statistical analyses were performed, as shown from 1 to 3 in the study workflow. Created with BioRender.com. **b**, **c** Graphics showing the relationship between the mean of (**b**) autoantibodies and (**c**) anti-SARS-CoV-2 levels in different age categories for healthy controls and COVID-19 disease groups. The size of the dots corresponds to the number of individuals (1–25 individuals) in the age category (see Supplementary Tables 1 and 2). The mean of the autoantibody levels by group is represented in natural logarithm. Error bars are S.E.M.

We employed a systems immunology approach (Fig. 1a) to investigate whether SARS-CoV-2 infection induces significant elevation of serum autoantibody levels in severe COVID-19 patients in an age-dependent manner. Figure 1b portrays the mean serum levels of all autoantibodies when comparing healthy individuals with mild, moderate, or severe COVID-19 patients. Meanwhile, the levels of anti-SARS-CoV-2 antibodies increased gradually from mild to severe disease (Fig. 1c; Supplementary Table 2).

**Fig. 2:**
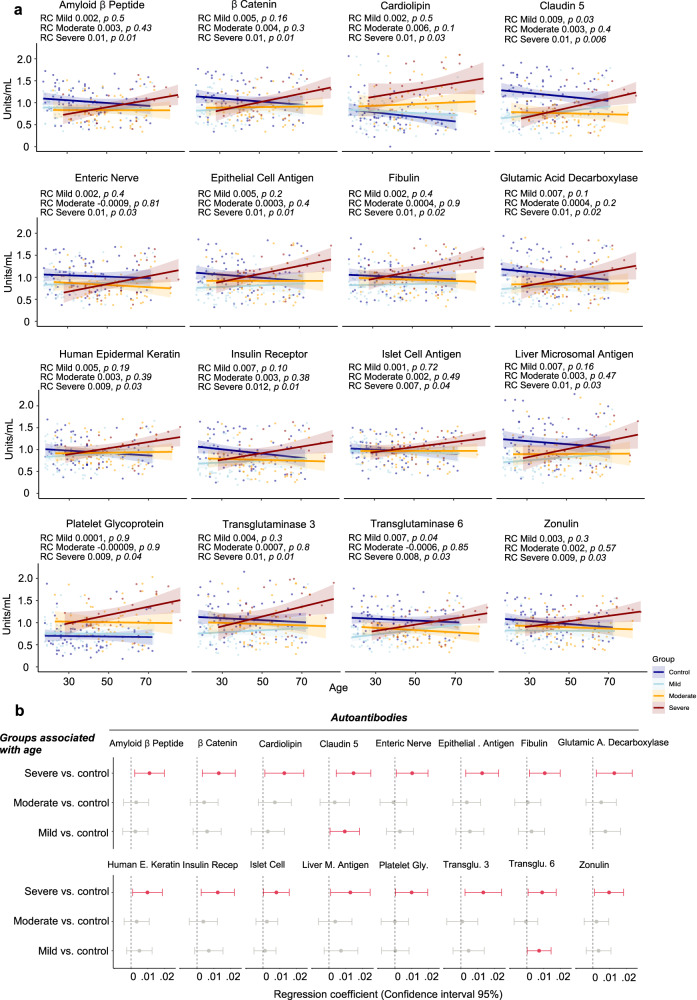
The relationship between autoantibodies and age in severe COVID-19 patients. **a** Scatter plot of regression analysis, indicating the relationship between the autoantibodies and age for COVID-19 and control groups. The *p*-values and multiple linear regression coefficients (RC) are displayed for each graph. Supplementary Table 4 shows the results of all regression coefficients. **b** Forest plots showing linear regression coefficients (dots) and their 95% confidence interval (whiskers) for different autoantibodies across the COVID-19 groups (mild, moderate, and severe) compared to healthy controls (vertical dotted line at the intercept of 0). Red dots and lines correspond to significantly increased autoantibody levels associated with disease group and age compared to healthy controls.

To characterize which autoantibodies significantly contributed to the age-associated enhancement of the autoantibody levels, we performed a multiple linear regression analysis for each autoantibody. Autoantibodies were the dependent variable, while group and age were the independent variables. In agreement with the descriptive statistical analysis shown in Fig. 1b, this inferential approach revealed autoantibodies targeting sixteen molecules strongly associated with age in the severe COVID-19 group compared with healthy controls. In contrast, the 95% confidence interval (CI) indicated a significant enhancement in the levels of autoantibodies targeting claudin 5 and transglutaminase 6 in the mild COVID-19 group but a non-significant range between higher and lower autoantibody levels for the mild and moderate COVID-19 groups when compared to healthy controls. (Fig. 2a, b; Supplementary Table 4). This indicates that many autoantibody levels robustly increase with age, particularly in severe COVID-19 patients, but lesser in mild and moderate COVID-19 patients.

### The effect of age on autoantibodies levels of COVID-19 patients

#### Different aging effects on autoantibodies from COVID-19 with age

**Fig. 3:**
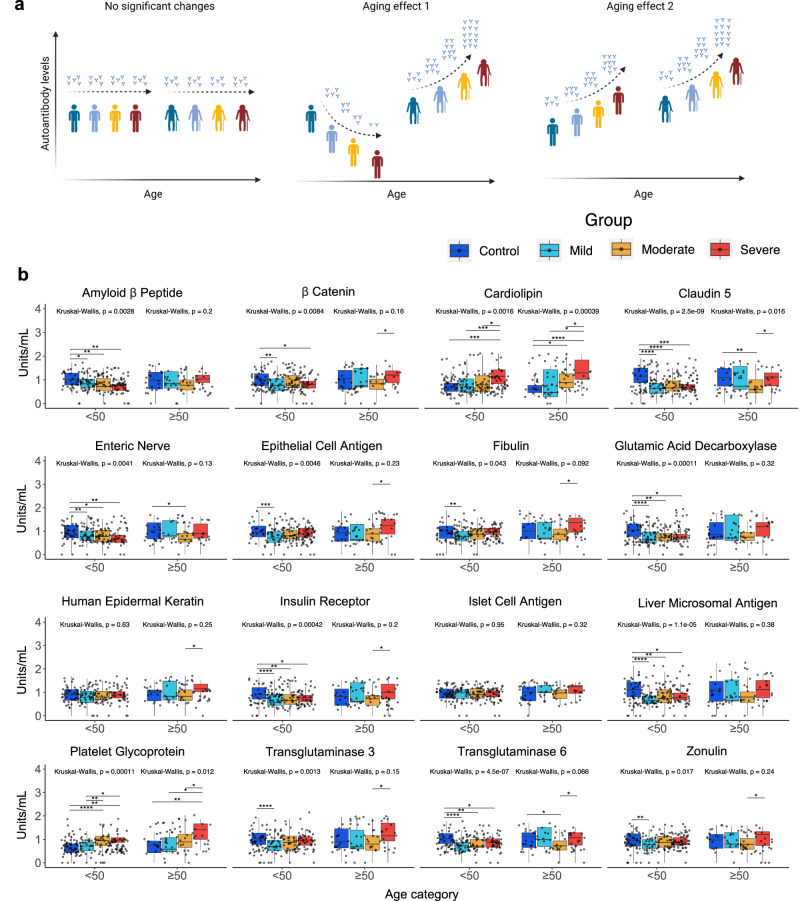
Aging effects on autoantibody levels according to COVID-19 severity. **a** Illustrative representations of aging effect patterns on autoantibody levels. From left to right: autoantibody levels not affected by age; reducing or increasing in COVID-19 patients <50 or ≥50 years old, respectively; or rising levels in both age groups as shown in detail in **b**. **b** Boxplots showing the autoantibody levels in young (<50 years old) and elderly (≥50 years old) groups for healthy controls as well as mild, moderate, and severe COVID-19 patients. The difference in autoantibody levels comparing young with elderly individuals of each group was calculated using the Kruskal Wallis and posthoc Dunn tests considering an FDR-adjusted *p*-value < 0.05 as significant (*p ≤ 0.05, **p ≤ 0.01, ***p ≤ 0.001). See Supplementary Table 5 for the exact numbers of *p*-values.

To further investigate the impact of age on the levels of autoantibodies, we divided the healthy controls and the COVID-19 patients into groups <50 or ≥50 years old for each category (healthy controls as well as mild, moderate, and severe COVID-19 patients). This approach revealed three overall patterns of autoantibody levels across the groups (Fig. 3a). Although some autoantibodies are significantly increased only in the severe COVID-19 patients ≥50 years old, we found in general comparable autoantibody levels (no significant aging effect) when analyzing healthy controls versus the COVID-19 subgroups; The first aging effect on the amount of autoantibodies was characterized by reduced levels with the COVID-19 severity, mainly in patients <50 years old, while increasing autoantibody levels in patients ≥50 years old according to the disease severity; on the other hand, the autoantibody levels increased according to the disease severity in individuals <50 or ≥50 years old, but more prominently in the latter group.

When compared to healthy controls, COVID-19 groups <50 years old exhibited a decreasing autoantibody level targeting amyloid β peptide, β catenin, claudin 5, enteric nerve, epithelial cell antigen, fibulin, glutamic acid decarboxylase, insulin receptor, liver microsomal antigen, transglutaminase 3, transglutaminase 6, and zonulin in accordance with COVID-19 severity (aging effect 1). Meanwhile, several autoantibodies increased in <50 years and ≥50 COVID-19 groups. However, only autoantibodies against cardiolipin and platelet glycoprotein significantly increased according to disease severity, regardless of age (aging effect 2) (Fig. 3b and Supplementary Table 5).

#### Autoantibodies associated with age stratify COVID-19 patients

**Fig. 4:**
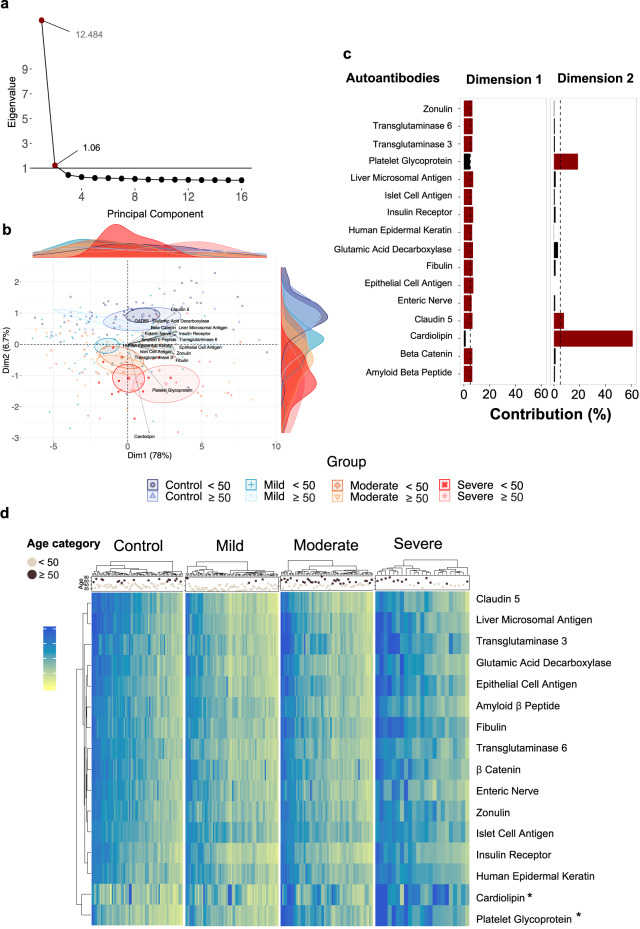
Autoantibodies linked to autoimmune diseases stratify COVID-19 severity by age. **a** Red dots show eigenvalues above one, and eigenvalues below one are shown by black dots demonstrating the importance of the dimensions (principal component). The horizontal black line shows the intercept of 1. Eigenvalues are available in Supplementary Table 6**. b** Barplots for two dimensions based on variable contribution. Each barplot shows the contribution (in %) of the sixteen autoantibodies to each dimension. The red coloured bars represent contribution values ≥ 5% (black dashed intercept line), while black coloured bars indicate contribution values < 5%. The contribution values of all autoantibodies to the different dimensions are listed in Supplementary Table 7. **c** PCA with spectral decomposition shows the stratification power of the sixteen most significant autoantibodies to distinguish between severe COVID-19 and healthy controls, considering the age categories of each group according to the first and second dimensions. **d** Heatmaps showing autoantibody levels ranging from 0 to 2 Units/ml according to the colour scale bar at the side of the graph clustered by Euclidian Distance for each disease and control group. The asterisk highlights the autoantibodies appearing as more elavated in severe COVID-19 patients. The age and age categories (light grey and brown dots above the heatmap for individuals <50 and ≥50 years old, respectively) for all individuals are shown above the heatmap. The bar ranging from yellow to blue (0 to 2) represents autoantibody levels in units/mL.

To investigate potentially age-related autoantibodies that stratify young from elderly severe COVID-19 patients and healthy controls, we carried out principal component analysis (PCA) based spectral decomposition^46^ (Fig. 4a–c). According to eigenvalue criteria, this may be viewed for just the first two dimensions (Intercept > 1; Fig. 4a). Thus, the PCA showed that healthy controls presented a similar autoantibody pattern independent of age category. In contrast, there was a gradual stratification of COVID-19 patients from mild to severe groups, which mapped most distantly from the healthy controls. In agreement with the results of the other analyses we performed, the aging impact was most evident when we compared severe COVID-19 <50 and ≥50 years old (Fig. 4b). Notably, the PCA with spectral decomposition indicated that autoantibodies targeting cardiolipin and platelet glycoprotein mainly contributed to dimension 2 (Fig. 4c), possibly being the autoantibodies mostly responsible for the stratification of severe COVID-19 patients <50 and ≥50 years old (Supplementary Tables 6 and 7).

Supporting the stratification power of autoantibody levels with age, hierarchical clustering analysis revealed similar results when comparing individuals <50 and ≥50 years old by disease severity. This approach uncovered a clear segregation of individuals <50 and ≥50 years old only in the severe COVID-19 patients but not the other groups investigated (Fig. 4d). This result suggests a more substantial aging effect on the severe COVID-19 group.

#### Ranking the age-associated autoantibodies that are most relevant for severe COVID-19

**Fig. 5:**
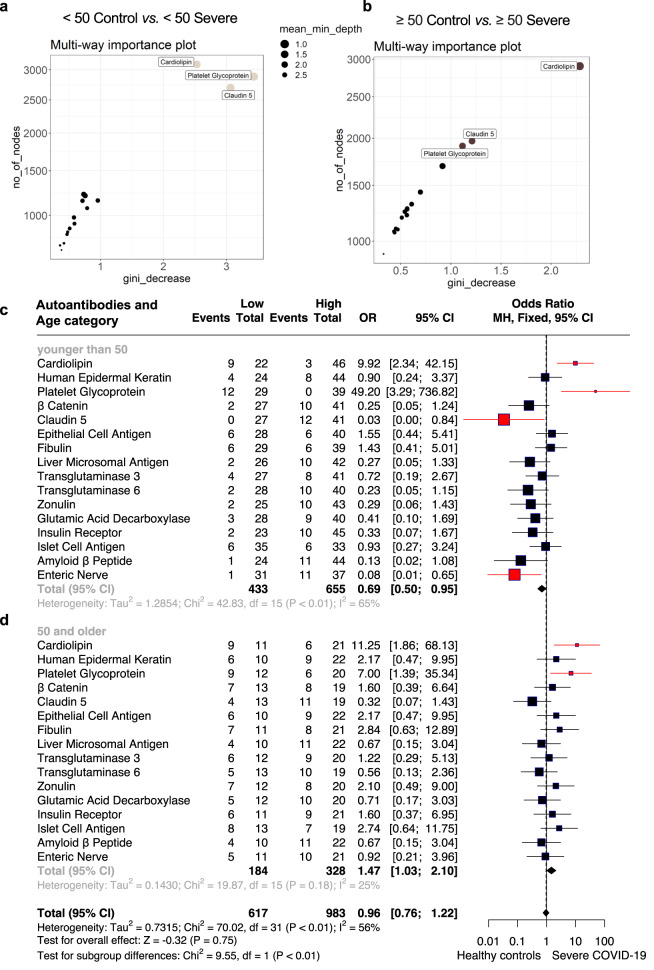
Ranking autoantibodies as predictors and classifiers of COVID-19 severity by age categories. **a**, **b** Random Forest model used to rank the 16 most essential autoantibodies as predictors for (**a**) severe COVID-19 <50 and (**b**) ≥50 severe COVID-19 patient groups compared to healthy controls. Multi-way importance plots show four nodes (IgG antibodies), the most significant predictors of severe COVID-19 <50 and ≥50. The size of the dots corresponds to the mean min depth in decreasing order (2.5 to 1.0). The names of the three most essential autoantibodies are shown by grey circles the <50 Control vs. <50 severe COVID-19 and in brown circles for the comparison ≥50 Control vs. ≥50 COVID-19 Severe. **c**, **d** Forest plot with an Odds ratio (OR) from binomial logistic regression analysis showing the regression coefficient (square) with confidence intervals (whiskers) and the significance level (≠1 intercept) for severe COVID-19 (**c**) <50 or (**d**) ≥50 compared to healthy controls. The exact values of OR and 95% confidence intervals are shown on the side of the regression coefficients. See Supplementary Fig. 2 for additional information. The red square indicates significant autoantibodies. Losango represents a Combined OR.

To better understand and identify the most relevant autoantibodies associated with severe COVID-19, we performed random forest analysis, comparing healthy controls versus severe COVID-19 patients (both groups comparing individuals <50 and ≥50 years old). This approach allows us to rank the most critical variables in a given dataset^47^: sixteen age-associated autoantibodies characteristic for COVID-19. This approach identified autoantibodies targeting cardiolipin, platelet glycoprotein, and claudin 5 as the three most essential autoantibodies classifying severe COVID-19 patients <50 and ≥50 years old when compared with healthy controls at these ages (Fig. 5a, b). The receiver operating characteristic (ROC) curves of these comparisons demonstrate the high accuracy of the random forest analysis based on the age-associated autoantibodies as classifiers of severe COVID-19 patients (Supplementary Fig. 1 and Supplementary Table 8).

The results suggest the importance of the autoantibodies targeting cardiolipin, platelet glycoprotein, and claudin 5 in the classification of COVID-19 patients. We conducted a binomial logistic regression analysis to better understand their contribution to developing severe COVID-19 disease. In this context, we also used a Linear discriminant analysis (LDA) model considering the groups (healthy controls versus severe COVID-19 patients (both groups ≥50 years old) as the dependent variables and the autoantibody levels as the independent variable to study the specificity and sensibility of age-associated autoantibodies targeting the 16 molecules to classify COVID-19 severity (Supplementary Tables 9 and 10). These approaches indicated only anti-cardiolipin and anti-platelet glycoprotein (when considering the sixteen age-associated autoantibodies) with specificity, sensitivity, and accuracy above 70% chance of a correct group classification (Supplementary Fig. 2). This approach allowed us to obtain the cut-off of specificity and sensibility of these autoantibodies. Furthermore, the Odds Ratio (OR) was calculated from the cut-off values obtained (Supplementary Table 11), allowing us to understand the relationship between the groups (healthy controls versus COVID-19 groups) as the dependent variable and the autoantibody levels as the independent variable to predict the likelihood of COVID-19 severity. Only anti-cardiolipin and anti-platelet glycoprotein showed a significantly increased odd ratio for severe COVID-19 patients <50 and ≥50 years old (Fig. 5c, d). On the other hand, following the aging effect 1 (Fig. 3), autoantibodies against claudin 5 and enteric nerve showed a significantly reduced OR in severe COVID-19 patients <50 in relation to healthy controls.

**Fig. 6:**
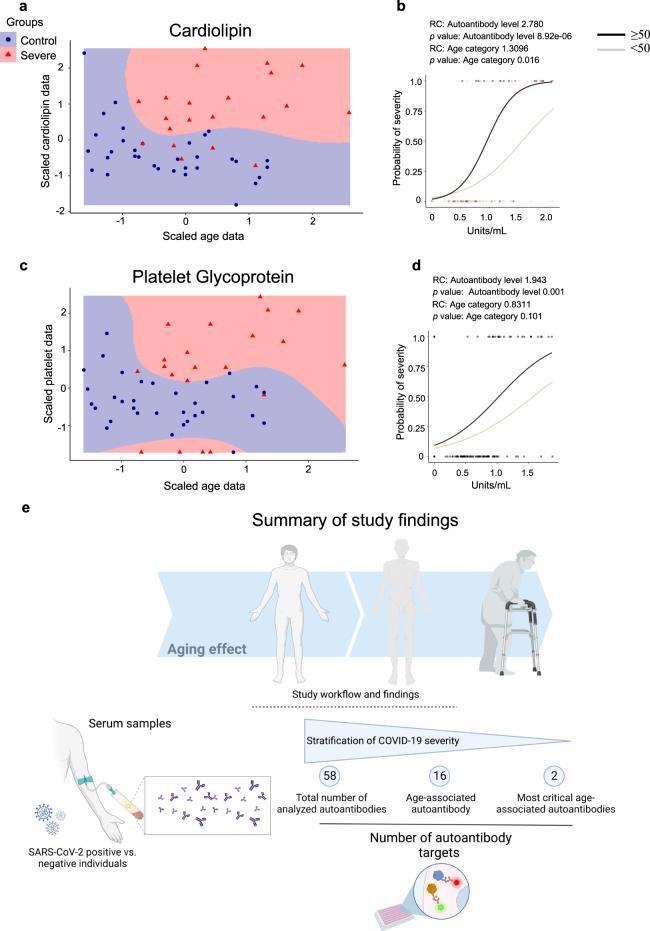
The probability of disease severity associated with anti-cardiolipin and anti-platelet autoantibody levels. Support Vector Machine (SVM, on the left) showing the non-linearly C-classification based on the radial kernel with over 70% (Supplementary Fig. 3 and Supplementary Table 10) accuracy between healthy controls and the severe COVID-19 group ≥50 years old. The scaled values for age (*x*-axis) of (**a, b**) anti-cardiolipin and (**c**, **d**) anti-platelet glycoprotein autoantibody levels (*y*-axis) are shown. The colours indicate each study group according to the figure legend. The scatter plot (on the right) of three autoantibodies (anti-cardiolipin and anti-platelet glycoprotein) shows that increased autoantibody levels can be explained by a higher probability of being severe in each group (healthy controls = 0, and severe COVID-19 = 1). The age category for each group is indicated by a light grey (<50 years old) and brown (≥50 years old) line in the graph. The regression coefficients to autoantibody levels and comparisons between the age categories are shown above each graph. **e** Summary of study findings. Created with BioRender.com.

Of note, the support vector machine (SVM) classification, which is a powerful machine learning approach with maximization (support) of separating margin (vector)^48,49^, based on the levels of anti-cardiolipin (Fig. 6a) or anti-platelet glycoprotein (Fig. 6c) in relation to age, confirmed the importance of these autoantibodies as suitable classifiers of severe COVID-19 when compared to healthy controls. I.e., SVM showed the separation of severe COVID-19 from healthy controls based on these most critical age-associated autoantibodies as the random forest analysis predicted. The input data are shown in Supplementary Tables 12 and Supplementary Fig. 3, while table results are exhibited in Supplementary Tables 13. In agreement, the binominal logistic regression indicated that the increase of anti-cardiolipin (Fig. 6b) or anti-platelet glycoprotein (Fig. 6d) levels enhance the probability of belonging to the severe COVID-19 group ≥50 years old when compared to those <50 years old.

## Discussion

Here we employed a systems biology approach to holistically understand the relationship between aging and the levels of serum autoantibodies linked to autoimmune diseases in patients with COVID-19. In line with the already well-known impact of patient aging, which is one of the most decisive risk factors for the development of severe COVID-19^50,51^ (in addition to other vital risk factors, such as obesity and prehistory of cardiovascular complications^52^), our data suggest that the production of natural autoantibodies (but not anti-SARS-CoV-2 antibodies) is significantly increased in an age-dependent manner, being most pronounced in individuals with severe COVID-19. Therefore, our work aligns with the seminal and outstanding reports from Bastard et al. and others who characterized that autoantibodies neutralizing type I IFNs are present in the general population and increase dramatically in prevalence after age 70, underlying 15-20% of cases of critical COVID-19^18,53,54^. These autoantibodies are also present in around 24% of breakthrough COVID-19 cases^55^, and are crucial risk factors for COVID death, especially in elderly individuals^51^. However, our data expand the number of age-associated autoantibodies and confirms the distinct impact of patient aging on the level of serum autoantibodies in severe COVID-19 patients, which we previously reported in patients with COVID-19^44,56,57^. We found that COVID-19 patients have a significant age-associated increase of autoantibody levels against 16 targets (e.g., amyloid β peptide, β catenin, cardiolipin, claudin, enteric nerve, fibulin, insulin receptor a, and platelet glycoprotein), which provides new avenues for mechanistic validation of these targets within the clinical context of their pathophysiology. Broadening the pool of targets may also provide a more comprehensive picture of the underlying pathophysiology of COVID-19 disease progression with advanced aging and, thus, improved targeting of suitable interventions.

Hierarchical clustering analysis of autoantibody levels indicated segregation of <50 from ≥50 years old patients with severe COVID-19. The combination of different machine learning approaches revealed that, among the significantly age-associated autoantibodies, particularly those directed against cardiolipin and platelet glycoprotein, are the most critical autoantibodies for predicting the severity of COVID-19 in older patients when compared to older healthy controls. Importantly, our data indicate a distinct separation/stratification of COVID-19 patients <50 from ≥50 years old and an increased OR of disease severity due to high levels of autoantibodies targeting cardiolipin and platelet glycoprotein. Indeed, the prothrombotic anti-cardiolipin autoantibodies that may potentially exacerbate the thrombo-inflammatory state related to severe COVID-19^21,58^, and other autoantibodies linked to classic autoimmune diseases^31^, have long been known to be highly prevalent in the healthy elderly population^59,60^.

Multiple linear and binominal logistic regression analyses indicated that autoantibodies targeting cardiolipin and platelet glycoprotein synergistically increase the probability of developing severe disease. Thus, in addition to the impaired immune response (affecting IFN-mediated immunity) and the generation of anti-type I IFN autoantibodies that drive the age-dependent severity of COVID-19^18,51,53,61^, patients with life-threatening SARS-CoV-2 infections also present with an age-dependent increase of multiple autoantibodies associated with classic autoimmune diseases that correlate with disease severity. The well-documented observation that anti-cardiolipin^62^ and anti-platelet antibodies^63^ increase the risk of thrombosis-related events such as pulmonary thromboembolism and deep vein thrombosis^64,65^ is also true for COVID-19 patients^11,21,24,66–68^. Thus, our findings could provide new insights into the complex pathophysiology of COVID-19, such as the thrombosis-related pathological events occurring with increased frequency in elderly individuals with SARS-CoV-2 infection. However, this represents a limitation of our study since we have no longitudinal data of our patients to evaluate if the individuals with high levels of anti-cardiolipin and anti-platelet antibodies subsequently developed thrombosis-related events.

Noteworthy, autoantibodies have been detected in healthy individuals at physiological levels^43,56,57,69–71^, are conserved among species and influenced by age, sex, and disease conditions^43^, and form network signatures^57^, including for instance, those targeting cardiolipin^59^ and platelet glycoprotein^45^. Based on our current and previous findings^57^, we postulate that autoantibodies are natural body components found at low or high levels in different autoimmune diseases^56^, and may pre-exist to pathological conditions. Hence, the elevated autoantibody levels associated with severe COVID-19 may be exacerbated by the evolutionarily conserved tendency to produce more autoantibodies with increasing age^42^. This phenomenon can aggravate the age-associated deficit in cardiovascular structure and function^72^ as well as the age-related decline of normal lung function^73^, which represent two central physiological systems (circulatory and respiratory) that are predominantly harmed in COVID-19 patients^74^. Together, these age-associated conditions create a fertile milieu for the poor outcomes of elderly individuals suffering from severe SARS-CoV-2 infection.

Considering the sequence of the underlying events, our results raise the critical question: does the severity of COVID-19 increase autoantibody levels? Or do the increased autoantibody levels affect the disease severity? We hypothesize that both possibilities are reasonable and may be complementary (i.e., bidirectional). The severe COVID-19 infection promotes a body environment such as tissue injury (acute respiratory distress syndrome or ARDS), cytokine storm, and macrophage hyperactivation^75^, which foster the production of autoantibodies. In turn, this disease context could allow those autoantibodies to act synergistically with multiple metabolites^76^, cytokines, and chemokines, which are naturally dysregulated in elderly patients as part of immunosenescence^35–37^, worsening the COVID-19 outcomes through several well-known mechanisms of autoantibody-induced pathology^77^. In this context, autoantibodies, in concert with other immune molecules (e.g., cytokines and chemokines), could interact in a highly complex network underlying immunopathological processes^78^ in severe COVID-19 patients, potentiated by aging-associated health conditions and lead to the development of severe disease.

Another age-dependent phenomenon that possibly explains the increased autoimmune responses we observed in the elderly patients with severe COVID-19 relies on the accumulation of epigenetic alterations (e.g., DNA methylation and histone acetylation)^79^, known to contribute to the autoimmunity risk of elderly individuals. Accordingly, accelerated epigenetic aging has been associated with the increased risk of SARS-CoV-2 infection and the development of severe COVID-19^80^. Lastly, a state of hyper-stimulation of the immune system by the SARS-COV-2 infection has been observed in elderly patients, for instance, by promoting the activation of overlapping B cell pathways between severe COVID-19 and patients with systemic autoimmune diseases^81^. Thus, several age-associated immunopathological events support the existence of age-associated autoantibodies, increasing the likelihood of severe COVID-19 disease in elderly patients.

In conclusion, our data provide new crucial insights into the critical relationship between severe COVID-19 and the increased dysregulation/production of distinct autoantibodies with increasing age that may be an essential component associated with developing severe COVID-19. As demonstrated by the stratification of young from elderly COVID-19 patients and the increased odds ratio of disease severity due to the high levels of autoantibodies linked to autoimmune diseases, in particular, those targeting cardiolipin or platelet glycoprotein, our data indicate an age-dependent effect of autoantibodies in the development of severe COVID-19, that may be of future value for disease prognosis. This work expands the link between senescence and aging with severe SARS-CoV-2 infection^82–87^.

However, our findings have limitations that require further investigation to fully understand the relationship between anti-cardiolipin and anti-platelet autoantibodies and COVID-19 severity. In addition to validating our results using another immunoassay platform and functional validation assays, these autoantibodies can also be present in individuals without COVID-19^59,66^. Thus, their presence alone does not necessarily indicate a severe or life-threatening disease^88^. However, there are some suggestions that anti-cardiolipin and anti-platelet autoantibodies may play a role in severe COVID-19. Studies^21,89^ have shown that COVID-19 patients with severe disease have higher levels of anti-cardiolipin autoantibodies than patients with milder disease or healthy controls. It has been postulated that these autoantibodies contribute to hypercoagulation and thrombosis in severe COVID-19, as they are associated with an increased risk of blood clots. Likewise, there is evidence^68^ suggesting that anti-platelet autoantibodies play a role in the hypercoagulability and thrombotic complications observed in severe COVID-19 patients. It has been reported that COVID-19 patients with severe disease had higher levels of anti-platelet factor 4 (PF4) antibodies than patients with milder disease or healthy controls. The study suggests^68^ that anti-PF4 antibodies in COVID-19 patients contribute to the hypercoagulability and thrombotic complications observed in severe disease. Therefore, future studies are needed to clarify the relationship between these autoantibodies’ presence, including these pro-thrombotic autoantibodies (i.e., anti-cardiolipin and anti-platelet glycoprotein) with COVID-19 outcomes (survival versus non-survival patients). In this context, it will be essential to evaluate the correlation between the level of autoantibodies according to aging (young versus elderly individuals) and other molecular measurements associated with higher COVID-19 risk mortality, such as T1/T2 cytokine profile, inflammatory markers in the populations with varying severity (defined by hospitalization and of days with fever).

Another limitation of our study is that we could not measure the autoantibody levels of our patient cohort before the SARS-CoV-2 infection (which is one of the fundamental constraints of many studies). This fact precludes us from determining a fundamental difference between autoantibodies that pre-exist SARS-CoV-2 infection and those that cause severe disease from autoantibodies triggered by infection (and are unlikely to cause or mitigate severe illness because of their delayed appearance). Thus, we cannot reject the possibility that some of our patients already had elevated levels of age-associated autoantibodies before developing severe COVID-19. Therefore, our findings could be influenced by this additional predisposition factor and its association with age and severe COVID-19.

## Methods

### Study cohort

We investigated 232 unvaccinated adults from the United States^44,90,91^, 159 COVID-19 patients with SARS-CoV-2 positive test by nasopharyngeal swab and polymerase chain reaction (PCR), and 73 randomly selected age—and sex-matched healthy controls who were SARS-CoV-2 negative by PCR and did not present any COVID-19 symptoms. COVID-19 patients were classified based on the World Health Organization (WHO) severity classification^92^ as mild COVID-19 (*n* = 71; fever duration ≤1 day; peak temperature of 37.8 C), moderate COVID-19 (*n* = 61; fever duration ≥7 days; peak temperature of ≥ 38.8 C), and severe COVID-19 patients (*n* = 27; severe symptoms and requiring supplemental oxygen therapy). All healthy controls and patients provided informed written consent to participate in the study following the Declaration of Helsinki. The study was approved by the IntegReview institutional review board (Coronavirus Antibody Prevalence Study, CAPS-613) and followed the reporting guidelines of Strengthening the Reporting of Observational Studies in Epidemiology (STROBE) (see demographic and clinical data in Supplementary Table 0).

### Measurements of anti-SARS-CoV-2 antibodies and autoantibodies linked to autoimmune diseases

Since the autoimmunity phenomenon has been linked with the severe acute respiratory syndrome coronavirus 2 (SARS-CoV-2), suggesting that COVID-19 patients can display features similar to a systemic autoimmune disease^19,93,94^, we decided to perform a comprehensive assessment of the influence of aging on the levels of autoantibodies linked to diverse autoimmune diseases. We included the time of sample collection in Supplementary Table 0. Since the COVID-19 groups presented a similar average of sample collection date, we excluded this variable as a potential confounder of patient subgroup comparisons.

Sera were assessed for the levels/titers of IgG anti-SARS-CoV-2 (Supplementary Table 1) antibodies (Catalogue number 109-055-008, Alkaline Phosphatase-conjugated AffiniPure Goat Anti-Human IgG, Fcγ Fragment Specific.) to spike and nucleocapsid proteins using the ZEUS SARS-CoV-2 ELISA Test System according to the manufacturer’s instructions (ZEUS Scientific, New Jersey, USA), as previously described^95^. We evaluated serum IgG autoantibodies against the nuclear antigen (ANA), extractable nuclear antigen (ENA), double-stranded DNA (dsDNA), actin, mitochondrial M2, and rheumatoid factor (RF) using commercial ELISA kits obtained from INOVA Diagnostics (San Diego, CA, USA). Furthermore, blinded, we quantified IgG autoantibodies against 52 target molecules using an in-house ELISA procedure (Immunosciences Lab., Inc; Los Angeles, CA USA). One hundred mL of each autoantigen at the optimal concentration were prepared in 0.01 M PBS pH 7.4 and aliquoted into microtiter plates. We used a set of plates and coated each well with 2% bovine serum albumin (BSA) or human serum albumin (HSA) as controls. The ELISA plates were incubated overnight at 4 °C and washed five times with 250 ml of 0.01 M PBS containing 0.05% Tween 20 pH 7.4. We avoided the non-specific binding of immunoglobins by adding 2% BSA in PBS and incubating the plates overnight at 4 °C. The plates were washed, and the serum samples from healthy controls and SARS-CoV-2 patients were diluted 1:100 in serum diluent buffer or 1% BSA in PBS containing 0.05% Tween 20 and incubated for 1 h at room temperature. The plates were rewashed, followed by the addition of alkaline phosphatase-conjugated goat anti-human IgG F(ab,)2 fragments (KPI, Gaithersburg, MD, USA) at an optimal dilution of 1:600 in 1% BSA PBS. The plates were incubated for an hour at room temperature and washed five times with PBS-Tween buffer. The enzyme reaction was started by adding 100 mL of para-nitrophenyl phosphate in 0.1 mL diethanolamine buffer 1 mg/mL plus 1 mM MgCl2 and sodium azide pH 9.8. Forty-five minutes later, the reaction was stopped with 50 mL of 1 N NaOH. The optical density (OD) was read at 405 nm using a microtiter plate reader. To exclude non-specific binding, the ODs of the control wells containing only HSA or BSA, always <0.15, were subtracted from those wells containing patient or control serum. The ELISA index for each autoantibody was calculated.

### Descriptive statistical analysis

We performed descriptive statistical analysis to demonstrate differences in the mean autoantibody levels. From this, we calculate the mean for each study group, using natural log for the comparisons. This analysis was performed using R^96,97^ programming version 4.2.1 (https://www.r-project.org/) and RStudio Version 2022.07.1 + 554^98^ (R package ggplot2^99^). To calculate the mean levels of autoantibodies, we used the R package stats^96,97^.

### Multiple linear regression

To further explore the relationship between the variables age and specific autoantibody levels in each study group, we applied multiple linear regression analysis^100^. This method evaluates the influence of age and study group on distinct antibody levels, thus, allowing to assess the relationship between the levels of autoantibodies or the levels of anti-SARS-CoV-2 with age as a continuous variable of the study group (healthy control; mild, moderate, and severe COVID-19). Our regression model was estimated based on all 59 (58 targets for autoantibodies plus the anti-SARS-CoV-2 antibody) explanatory variables. R programming was used to divide multiple R-squared by each explanatory variable’s residual standard error (sigma). After that, the effect size f2 was obtained using the G-power software^101^. This approach allowed us to calculate the sample size required for each explanatory variable (Supplementary Table 3). We found that autoantibodies against seven molecules did not fit our sample size. Thus, they were already excluded from the initial manuscript version. Therefore, the sample size of our study was statistically appropriately used in the linear multiple regression model.

Furthermore, patients’ sex was considered a covariable in the regression model since it represents a confounder that may influence the dependent variables. We used the *lm* function from the R package stats^96,97^ for the multiple linear regression analysis, and forest plots and scatter plots were generated using the R package ggplot2^102^.

### Differences in autoantibody levels by age category

We used box plots to show the distribution levels of autoantibodies in healthy controls and each COVID-19 group (mild, moderate, and severe), classifying individuals <50 or ≥50 years of age^45^. Statistical differences in autoantibody levels were calculated using the Kruskal Wallis test followed by the posthoc Dunn test, considering *p*-value and adjusted *p*-value (False Discovery Rate [FDR]) <0.05 as the significance cut-off, respectively. Box plots were generated using the R packages rstatix^103^ and ggplot2^102^.

### Principal component analysis and hierarchical clustering

Based on the multiple linear regression results, we identified significantly increased titers of age-associated autoantibodies against 16 targets, which underwent PCA with spectral decomposition^104,105^, as previously described^44,106^. This approach allowed us to measure the stratification power of the autoantibodies in distinguishing between severe COVID-19 patients and healthy controls while considering young and elderly groups. We calculated the eigenvalues based on the contributions of autoantibody levels to demonstrate their direction in the principal component analysis. The eigenvalues and eigenvectors exceeding one intercept^107^ were considered essential to show the segregation of groups. For this, we used the R functions *get_eig* and *get_pca_var* from factoextra package^108^. PCA was performed using the function *prcomp* from the same package. Additional visualization of autoantibody levels in the different study groups was performed using the R package ComplexHeatmap^109^ and Circlize^110^. The clustering of autoantibody levels in each study group was based on Euclidian distance.

### Random forest modelling

We employed the random forest model to rank the most relevant autoantibodies (the autoantibodies that were significant in the multiple regression analysis) to best classify COVID-19 disease severity for each age category (<50 and ≥50 years old) using the R package randomForest (version 4.7.1.1)^47^ as previously described^31,44,111^. Briefly, five thousand trees were used, and three variables were resampled (mtry parameter). As criteria to determine variable importance in the classification, we considered the mean minimum depth, Gini decrease, and the number of appearances in nodes. The dataset was split into training and testing sets using a 3 to 1 ratio for cross-validation, while quality was assessed for each, respectively, through out-of-bags error rate and the ROC curve.

### Odds ratio to belong to the severe COVID-19 group

To calculate the OR to belong to the severe COVID-19 group based on autoantibody levels, we carried out LDA and logistic binomial regression. The LDA is a method to find a linear combination of variables (autoantibodies) that characterize two or more classes of objects/events^112^, herein, individuals healthy controls vs. severe COVID-19 <50 or ≥50 years old. Autoantibodies with a specificity and sensitivity value >70% were considered a threshold to belong to the severe COVID-19 groups <50 or ≥50 years old. Based on this threshold, we categorize the detection values of each autoantibody from 0 and 1 for both age categories. The analysis was performed using the R package MASS^113^ with the *lda* function. To plot the specificity and sensitivity of the class prediction for each autoantibody, we used the R package plotROC^114^ and ggplot2^102^. In addition, we performed the binomial logistic regression for the OR^115^ from the LDA results to predict COVID-19 severity using the function *logistic.display* of the R package epiDisplay^116^. Plots resulting from this analysis were generated using the R package meta^117^.

### Support Vector Machine (SVM) classification and probability by binomial logistic regression

We used^118,119^ SVM, a robust computer algorithm, to build classifiers^48^. SVM employs four basic concepts: separating hyperplane, the maximum-margin hyperplane, the soft margin, and the kernel function^49^. We performed the radial kernel function applied between healthy controls and the severe COVID-19 group to classify the scaled values of the anti-cardiolipin and anti-platelet glycoprotein autoantibodies with age. Groups were defined as the dependent variable, while antibodies and age were considered independent variables. The analysis was performed using the *svm* function of the e1071^120^ R package. We used the kernel (C-classification) with 50% of our data sorted randomly by the R base *sample* function for training and predicting, considering the *radial basis* parameter, the best model applied to our data. Accuracy was defined as the percentage of correctly classified samples resulting in 77% for cardiolipin and 81% for platelet glycoprotein, correctly classified as healthy controls and severe COVID-19 patients in our model. Furthermore, we used the *tune* function of the R package e1071^120^ to adjust the hyperparameters for cost and gamma in the *svm* function. We used a cost of 10 and a gamma of 0.5 for our data. All graphs from *svm* prediction results were generated using the R package ggplot2^102^. In addition, we used the binomial logistic regression analysis to understand whether the severity of COVID-19 can be predicted based on age and autoantibody levels. The binomial logistic regression analysis indicates the probability that an observation falls into one of two defined dichotomous categories based on one or more independent variables^115^. This analysis was performed using the R package stats^97^ with the *glm* function. The categories of the dichotomous dependent variable were defined as “belonging to severe COVID-19: group 1” and “not belonging to severe COVID-19: group 0”, using the binomial logistic family to predict the probability of falling into the severe COVID-19 group in relation to the healthy controls. This approach resulted in a regression coefficient and *p*-value for the probability of severe COVID-19 based on the autoantibody level and the likelihood of severe COVID-19 based on the age category.

### Reporting summary

Further information on research design is available in the Nature Research Reporting Summary linked to this article.

### Supplementary information

Supplementary Tables

Supplementary Figures

Reporting Summary

## Data Availability

All data generated in this study are provided in the Supplementary Data. All input and output data, such as supplementary tables data files, are provided. Primary input data is available in Supplementary Table 1.

## Code availability

All R packages used in this manuscript are described in the Reporting Summary and are available at the following link: https://github.com/Dennyson83/A-systems-biology-approach.
