# Supplementary material for: Severe COVID-19 patients exhibit elevated levels of autoantibodies targeting cardiolipin and platelet glycoprotein with age: a systems biology approach
Source: NPJ Aging. 2023 Aug 24;9(1):21. doi: 10.1038/s41514-023-00118-0 (PMC10449916; doi:10.1038/s41514-023-00118-0)
Supplement: Supplementary file 2 — Supplementary Figures [file 41514_2023_118_MOESM2_ESM.pdf]

Supp Figure 1

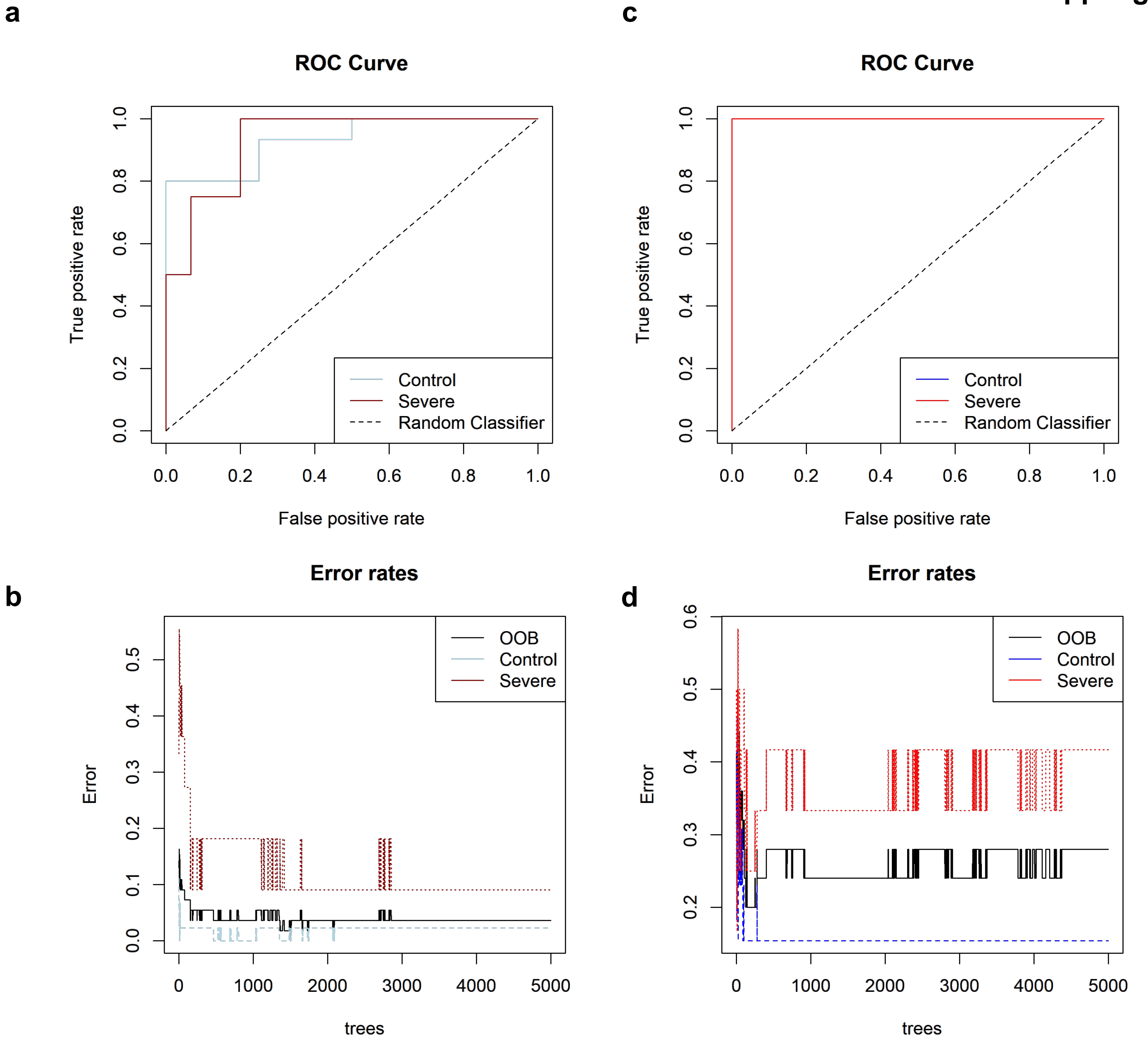

Supplementary Figure 1. a-b) ROC curve of the random forest model showing over 70% specificity and sensitivity for healthy controls and severe COVID-19 groups of each age category (young and elderly). **c-d)** Stable curve showing the number of trees and out-of-bag (OOB) error rate of 20% for young healthy controls and 9% for the young severe COVID-19 group (left side graph of the figure), as well as an error rate of 15% for elderly healthy controls and 41% for the elderly severe COVID-19 group (right side graph of the figure). **Supplementary Table 5** shows the confusion matrix for the random forest model.

Supp Figure 2

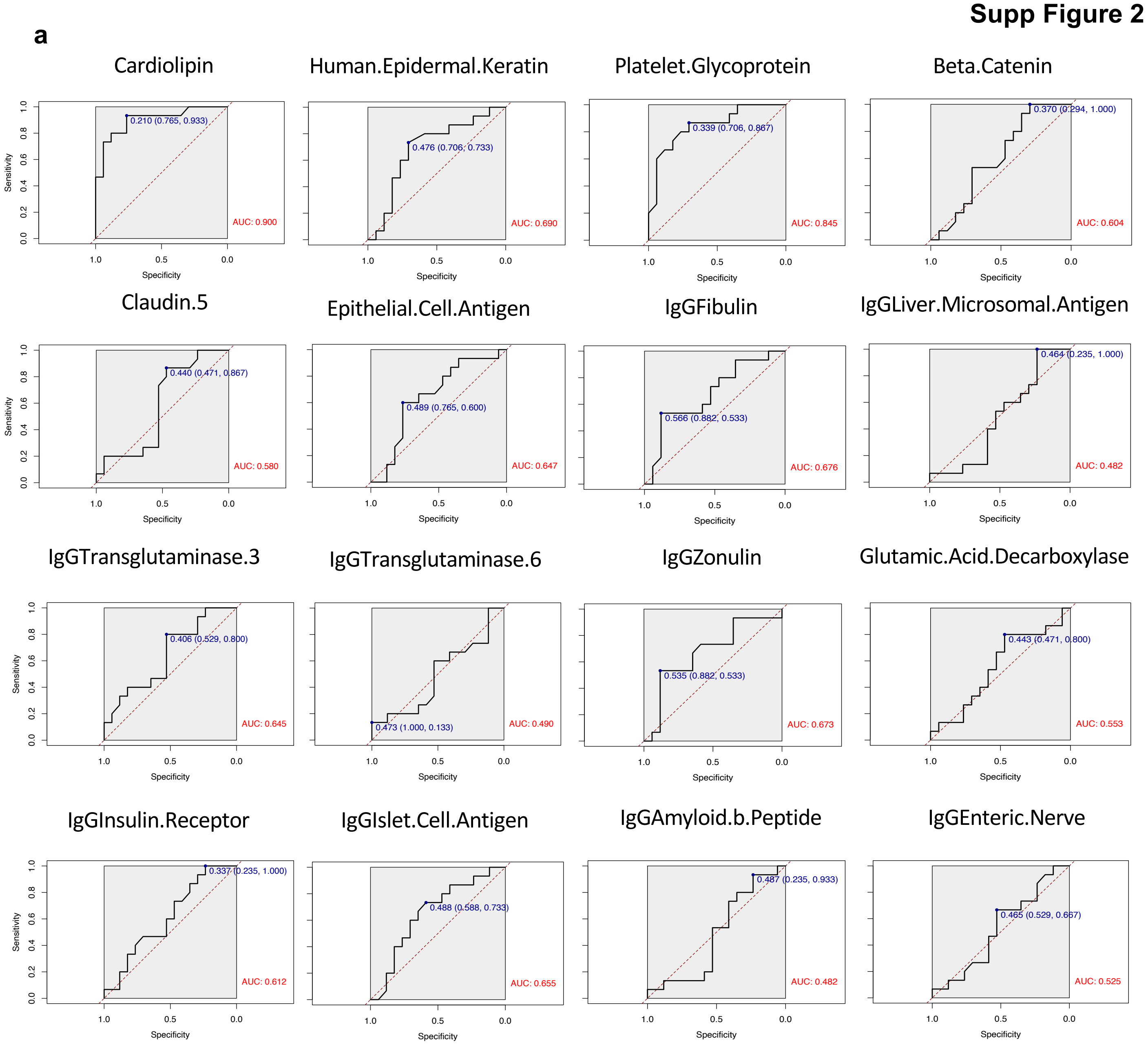

Supplementary Figure 2. ROC curves demonstrate the specificity and sensitivity of the sixteen autoantibodies for severe COVID-19 in the elderly category. All plots were generated using binomial logistic regression.

Supp Figure 3

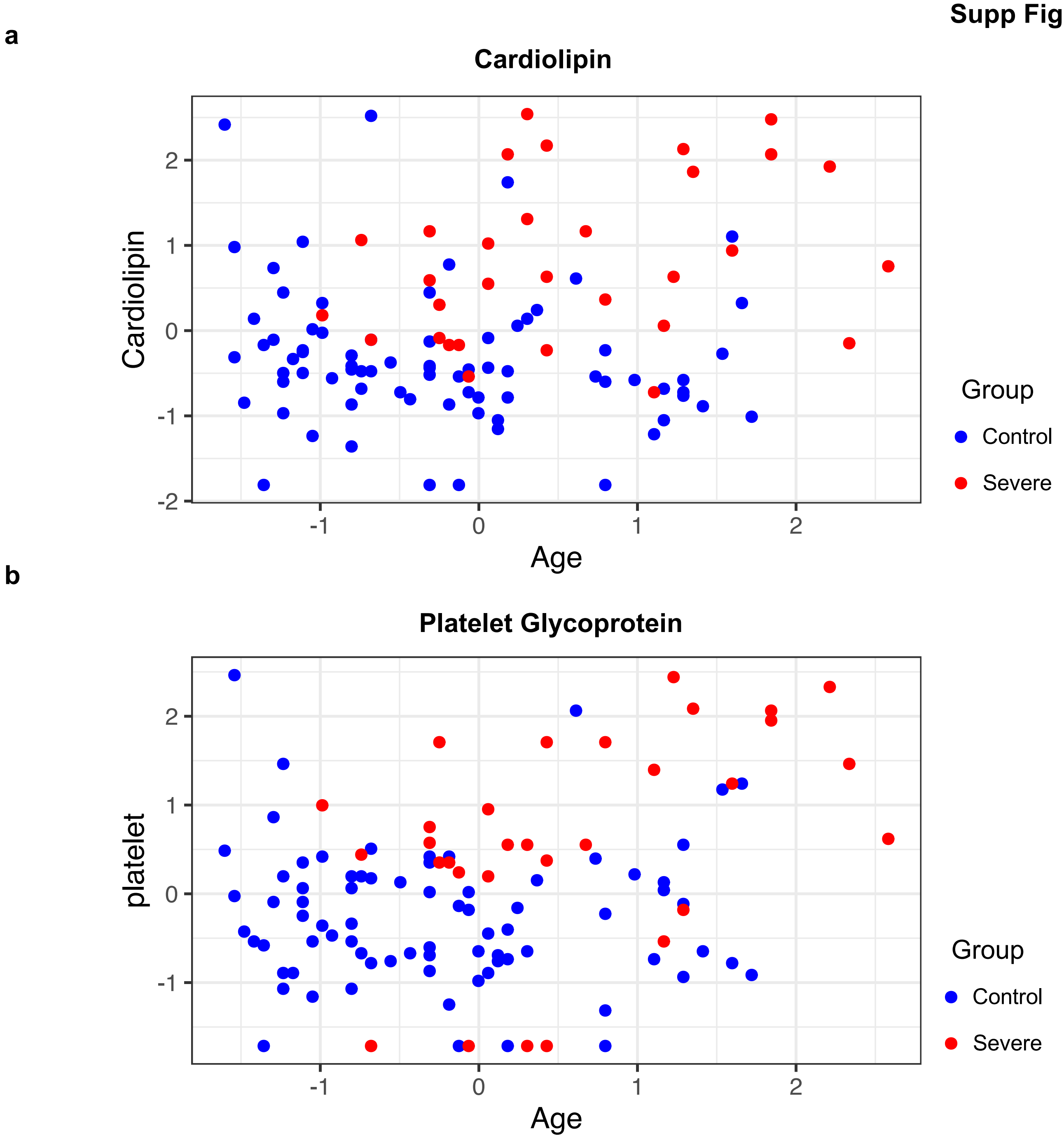

Supplementary Figure 3. a-b) Scatter plots showing the distribution of elderly individuals in the healthy control (blue dots) and severe COVID-19 group (red dots) for (**a**) anti-cardiolipin antibody levels and (**b**) anti-platelet glycoprotein levels. This distribution was used as input for the SVM analysis.
