# Supplementary material for: Severe COVID-19 patients exhibit elevated levels of autoantibodies targeting cardiolipin and platelet glycoprotein with age: a systems biology approach
Source: NPJ Aging. 2023 Aug 24;9(1):21. doi: 10.1038/s41514-023-00118-0 (PMC10449916; doi:10.1038/s41514-023-00118-0)
Supplement: Supplementary file 3 — Reporting Summary [file 41514_2023_118_MOESM3_ESM.pdf]

nature portfolio

Corresponding author(s): NPJAMD-00492-T

Last updated by author(s): 05/18/2023

Reporting Summary

Nature Portfolio wishes to improve the reproducibility of the work that we publish. This form provides structure for consistency and transparency in reporting. For further information on Nature Portfolio policies, see our Editorial Policies and the Editorial Policy Checklist.

Statistics

For all statistical analyses, confirm that the following items are present in the figure legend, table legend, main text, or Methods section.

n/a	Confirmed	
☐	☒	The exact sample size (n) for each experimental group/condition, given as a discrete number and unit of measurement
☐	☒	A statement on whether measurements were taken from distinct samples or whether the same sample was measured repeatedly
☐	☒	The statistical test(s) used AND whether they are one- or two-sided<br>*Only common tests should be described solely by name; describe more complex techniques in the Methods section.*
☐	☒	A description of all covariates tested
☐	☒	A description of any assumptions or corrections, such as tests of normality and adjustment for multiple comparisons
☐	☒	A full description of the statistical parameters including central tendency (e.g. means) or other basic estimates (e.g. regression coefficient) AND variation (e.g. standard deviation) or associated estimates of uncertainty (e.g. confidence intervals)
☐	☒	For null hypothesis testing, the test statistic (e.g. F, t, r) with confidence intervals, effect sizes, degrees of freedom and P value noted<br>*Give P values as exact values whenever suitable.*
☒	☐	For Bayesian analysis, information on the choice of priors and Markov chain Monte Carlo settings
☐	☒	For hierarchical and complex designs, identification of the appropriate level for tests and full reporting of outcomes
☐	☒	Estimates of effect sizes (e.g. Cohen's d, Pearson's r), indicating how they were calculated

Our web collection on statistics for biologists contains articles on many of the points above.

Software and code

Policy information about availability of computer code

Data collection	The ELISA data obtained (autoantibodiy levels) are described in the material e methods and provided as supplementary files.
Data analysis	R programming version 4.2.1 (https://www.r-project.org/) and RStudio Version 2022.07.1+55499 (R package ggplot2100).

For manuscripts utilizing custom algorithms or software that are central to the research but not yet described in published literature, software must be made available to editors and reviewers. We strongly encourage code deposition in a community repository (e.g. GitHub). See the Nature Portfolio guidelines for submitting code & software for further information.

Data

Policy information about availability of data

All manuscripts must include a data availability statement. This statement should provide the following information, where applicable:

- Accession codes, unique identifiers, or web links for publicly available datasets
- A description of any restrictions on data availability
- For clinical datasets or third party data, please ensure that the statement adheres to our policy

All data generated in this study are provided in the Supplementary Data. All input and output data, such as supplementary tables data files, are provided. Primary input data is available as supplementary table S1

Research involving human participants, their data, or biological material

Policy information about studies with human participants or human data. See also policy information about sex, gender (identity/presentation), and sexual orientation and race, ethnicity and racism.

Reporting on sex and gender	We investigated 232 unvaccinated adults from the United States44,91,92, 159 COVID-19 patients with SARS-CoV-2 positive test by nasopharyngeal swab and polymerase chain reaction (PCR), and 73 randomly selected age - and sex-matched healthy controls who were SARS-CoV-2 negative by PCR and did not present any COVID-19 symptoms.
Reporting on race, ethnicity, or other socially relevant groupings	We investigated 232 unvaccinated adults from the United States44,91,92, 159 COVID-19 patients with SARS-CoV-2 positive test by nasopharyngeal swab and polymerase chain reaction (PCR), and 73 randomly selected age - and sex-matched healthy controls who were SARS-CoV-2 negative by PCR and did not present any COVID-19 symptoms.
Population characteristics	COVID-19 patients were classified based on the World Health Organization (WHO) severity classification93 as mild COVID-19 (n=71; fever duration ≤ 1 day; peak temperature of 37.8 C), moderate COVID-19 (n=61; fever duration ≥ seven days; peak temperature of ≥ 38.8 C), and severe COVID-19 patients (n=27; severe symptoms and requiring supplemental oxygen therapy). All healthy controls and patients provided informed written consent to participate in the study following the Declaration of Helsinki. The study was approved by the IntegReview institutional review board (Coronavirus Antibody Prevalence Study, CAPS-613) and followed the reporting guidelines of Strengthening the Reporting of Observational Studies in Epidemiology (STROBE) (see demographic and clinical data in Supplementary Table S0).
Recruitment	Patients and controls were recruited through a survey including 81 data points including questions about patient demographics, contacts with other Covid-19–infected individuals in the household, symptoms of SARS-CoV-2, whether they tested positive for SARS-CoV-2 by nasal swab (yes/no), and required oxygen for SARS-CoV-2 throughout their illness. The survey was administered via the Health Insurance Portability and Accountability Act-compliant and secure Research Data Capture software
Ethics oversight	The study was approved by the IntegReview institutional review board (Coronavirus Antibody Prevalence Study, CAPS-613) and followed the reporting guidelines of Strengthening the Reporting of Observational Studies in Epidemiology (STROBE)

Note that full information on the approval of the study protocol must also be provided in the manuscript.

Field-specific reporting

Please select the one below that is the best fit for your research. If you are not sure, read the appropriate sections before making your selection.

☒ Life sciences ☐ Behavioural & social sciences ☐ Ecological, evolutionary & environmental sciences

For a reference copy of the document with all sections, see nature.com/documents/nr-reporting-summary-flat.pdf

Life sciences study design

All studies must disclose on these points even when the disclosure is negative.

Sample size	the effect size f2 was obtained using the G-power software102. This approach allowed us to calculate the sample size required for each explanatory variable (Supplementary Table S3). Therefore, the sample size of our study was statistically appropriately used in the linear multiple regression model.
Data exclusions	We found that autoantibodies against seven molecules did not fit our sample size. Thus, they were already excluded from the initial manuscript version.
Replication	Here we performed a cross sectional study, including 232 unvaccinated adults from the United States44,91,92, 159 COVID-19 patients with SARS-CoV-2 positive test by nasopharyngeal swab and polymerase chain reaction (PCR), and 73 randomly selected age - and sex-matched healthy controls who were SARS-CoV-2 negative by PCR and did not present any COVID-19 symptoms.
Randomization	IgG autoantibodies against 52 target molecules were quantified in a blinded fashion, using an in-house ELISA procedure.
Blinding	IgG autoantibodies against 52 target molecules were quantified in a blinded fashion, using an in-house ELISA procedure.

Reporting for specific materials, systems and methods

We require information from authors about some types of materials, experimental systems and methods used in many studies. Here, indicate whether each material, system or method listed is relevant to your study. If you are not sure if a list item applies to your research, read the appropriate section before selecting a response.

Materials & experimental systems

n/a	Involved in the study
	☒ Antibodies
☒	☐ Eukaryotic cell lines
☒	☐ Palaeontology and archaeology
☒	☐ Animals and other organisms
	☒ Clinical data
☒	☐ Dual use research of concern
☒	☐ Plants

Methods

n/a	Involved in the study
☒	☐ ChIP-seq
☒	☐ Flow cytometry
☒	☐ MRI-based neuroimaging

Antibodies

Antibodies used	Sera were assessed for the levels/titers of IgG anti-SARS-CoV-2 (Supplementary Table S1) antibodies to spike and nucleocapsid proteins using the ZEUS SARS-CoV-2 ELISA Test System according to the manufacturer's instructions (ZEUS Scientific, New Jersey, USA)
Validation	Details are found in the following link: https://www.fda.gov/media/142809/download

Clinical data

Policy information about clinical studies

All manuscripts should comply with the ICMJE guidelines for publication of clinical research and a completed CONSORT checklist must be included with all submissions.

Clinical trial registration	PROTOCOL NUMBER AND TITLE OF STUDY: CAPS-613; "Coronavirus Antibody Prevalence Study (CAPS)"
Study protocol	Details of the study protocol have been previously published: https://www.sciencedirect.com/science/article/pii/S2213219821007790
Data collection	Subjects were recruited by local not-for-profit and social service organizations within orthodox Jewish communities across 5 states (California, Connecticut, Michigan, New Jersey, and New York) between May 13 and July 6, 2020.
Outcomes	The survey was developed to determine the most common symptoms and outcomes of SARS-CoV-2. The survey included 81 data points including questions about patient demographics, contacts with other Covid-19–infected individuals in the household, symptoms of SARS-CoV-2, whether they tested positive for SARS-CoV-2 by nasal swab (yes/no), and required oxygen for SARS-CoV-2 throughout their illness. The survey was administered via the Health Insurance Portability and Accountability Act-compliant and secure Research Data Capture software.
